# How Do Participatory Citizenship Models Inform Co‐production in Health Research? A Systematic Review

**DOI:** 10.1111/hex.70394

**Published:** 2025-08-20

**Authors:** Catharine Rose, Lois Donnelly, Jess Howdle, Mark Lynes, Katharine Arnett, Mary Nettle, Eleanor Bradley

**Affiliations:** ^1^ Research Office University of Worcester Worcester UK

**Keywords:** citizenship, co‐production, health inequalities, patient and public participation, public involvement, systematic review, values

## Abstract

**Background:**

Co‐production within health research has increased rapidly over the past two decades, enabling citizens to shape health policy and practice. Yet, co‐production can be shallow or undemocratic, providing little opportunity for meaningful citizen involvement. Participatory citizenship models are frameworks that outline the criteria by which citizens to belong and participate in a society. This review aimed to identify how participatory citizenship is enabled through co‐production.

**Methods:**

A systematic review was conducted of studies in which a participatory citizenship model informed co‐production strategies in health research.

**Findings:**

Of 215 unique articles, 13 articles met the inclusion criteria and were included in the synthesis. Four aspects characteristic of how participatory citizenship models inform co‐production in health research were identified. These were, Aspect (1) *Co‐production enables diverse citizens to participate in health research*; Aspect (2) *Citizens′ lived‐experience contextualises and shapes all stages of the health research process*; Aspect (3) *Co‐production shares power and ownership between citizens and research teams;* and, Aspect (4) *Co‐production through health research enables citizens to actively participate in the development of their communities*. The first three aspects enable the fourth aspect to be fully enacted within a research project.

**Conclusion:**

Citizenship‐informed co‐production ensures research enables local citizens to apply their lived‐experience and local knowledge to shape community health and is valuable to policymakers and practitioners working to reduce health inequalities. Researchers are encouraged to engage with these four aspects through authentic co‐production. The authors present a set of recommendations for researchers based on the findings of the synthesis.

## 1 Introduction

### 1.1 Co‐production in Health Research

Through co‐production, citizens can be empowered to use lived‐experience to shape the health policies and services that impact their communities [1, 2]. Co‐production in research involves sharing power among those who produce research, participate in research, and who are impacted by research findings [3, 4]. Co‐production in health research has increased rapidly over the last decade, with most academic publications discussing co‐production originating from Anglo‐Saxon heritage countries, notably the UK [5, 6]. This reflects that the UK Health Service and its leading research funders, including the National Institute for Health Research, recommend or mandate co‐production, aligning the practice with service redesign and improvement [5, 7]. Although co‐production is often subsumed under the banner of Public and Patient Involvement and Engagement (PPIE), not all PPIE activities entail co‐production. ‘Engagement’ means public connection with research through dissemination, education or information. ‘Involvement’ may entail co‐production, where public members are actively involved in research production as *co‐researchers*, but also includes activities where public members are not part of the research team, including providing consultation responses, advice, or feedback to researchers [8, 9].

No single definition of co‐production exists, which is described as an ‘*elastic*’ concept [1, 10]. A broad definition of co‐production is ‘*joint working between people or groups who have traditionally been separated into categories of user and producer*’ [1]. There are many co‐production techniques, few parameters, and ad hoc contextual adjustments frequently occur in practice [1, 10]. Clark [10] describes co‐production as a contextual ‘*craft*’ developed through working with communities and through researchers' shared experiences. Ducrose et al [1] suggest the relatively weak evidence base for co‐production stems from the unsuitability of its flexible and contextual techniques for measurement within large‐scale, deductive, evaluation models. However, whilst the ‘craft’ of co‐production is inherently diverse and flexible, researchers need pragmatic guidance and tools to effectively co‐produce research with the public, and a plethora of frameworks, toolkits and guidance have been developed to meet this need [6, 11]. A systematic review by Greenhalgh et al. [6] identified 65 co‐production frameworks, mostly assessed as being of high quality, and most used only by the group which developed them. Greenhalgh et al. [6] recognised that existing frameworks had been developed to fit the variations of different fields and contexts reflecting the elasticity of co‐production noted by various authors [1, 10]. In response, Greenhalgh et al. [6] recommended that research teams co‐produce bespoke frameworks that draw on, but are not restricted by, existing tools [6].

### 1.2 Citizenship Models

Citizenship models are frameworks outlining the criteria by which citizens contribute to and belong to a society. Participatory citizenship models extend the social citizenship framework, which exchanges socially responsible actions for social rights, to involve citizens in creating solutions to social problems through activities like policy‐making and community development [12]. Traditional citizenship models skewed power towards those authorities defining the terms of citizenship, but contemporary citizenship models are characterised by more democratic power structures that empower citizens to play an authentic and active role in shaping society [12, 13, 14, 15]. In contemporary heterogeneous societies, civic participation involves a complex negotiation between intersecting identities (e.g., gender, race) and citizenships, as people become multi‐national, global, and digital citizens [12, 14, 15, 16].

The wider determinants of health reflect socioeconomic and environmental factors which shape people's health [17, 18]. Health research is designed to improve health outcomes and generate an understanding of how changes in these wider determinants influence individual, family, and community health. Through co‐production, citizens can actively engage with health research teams to develop together research ideas and outcomes which may positively shape health in their communities. Enacting citizenship through research participation may be particularly valuable for marginalised groups who are vulnerable to both civic exclusion and health inequalities [15, 19, 20]. Yet despite the potential, research shows that co‐production can be tokenistic, offering citizens little shared power or genuine agency [21, 22]. Given the heterogeneity of co‐production methods, the conceptualisation of co‐production would benefit from an appropriate theoretical underpinning relevant to the aims of co‐production. The current authors argue that participatory citizenship models serve as relevant theoretical underpinnings that see co‐production in health research as a mechanism by which to engage citizens with civic activity that shapes and benefits their communities. Existing co‐production frameworks are predominantly inwardly‐focused on the research group, context or study in focus [6]. Participatory citizenship models can provide an underpinning outward focus that engages citizens to contribute towards community development through and beyond the specific, time‐limited space of a research project. From this theoretical foundation, effective and transferable recommendations for researchers could be developed. As a first step, the current study aimed to identify, through the synthesis of co‐produced health research studies, how participatory citizenship models inform co‐production.

## 2 Materials and Methods

### 2.1 Design

A systematic review to synthesise co‐produced health research studies underpinned by a participatory citizenship model. The research team included three authors with lived‐experience of health services (ML, KA, MN). A systematic literature search process was followed, as outlined by the Preferred Reporting Items for Systematic Reviews and Meta‐Analyses (PRISMA) [23].

### 2.2 Protocol and Registration

The protocol was registered with OSF Registries on January 29, 2024, before the review began. This document was updated when changes were made to the protocol [24].

### 2.3 Inclusion and Exclusion Criteria

Articles were included in the review if they met the inclusion criteria detailed in Table 1.

**TABLE 1:** Inclusion and exclusion criteria.

Inclusion criteria	Exclusion criteria
Empirical studies	Non‐empirical work (e.g., reviews, editorials, essays, opinion pieces, conference presentations)
Research related to health care or health policy	Topics unrelated to health
Research underpinned by a participatory citizenship model, or synonymous model*	Work that is not underpinned by a participatory citizenship model, or synonymous model*
Researchers engaging co‐production with citizens	Research that is not co‐produced with citizens
All co‐production/research participants ≥ 18 years old	Co‐production/research participants are children or adolescents (< 18 years old); animal studies
Worldwide studies	Articles in languages other than English or Welsh (with no available English translation)
English or Welsh language articles (including translated articles)	Articles that are not peer‐reviewed, including grey literature
Peer‐reviewed articles	Postgraduate theses; articles not peer‐reviewed

* Participatory citizenship models empower citizens to engage in shaping local, national and/or global society.

### 2.4 Search Strategy

**FIGURE 1:**
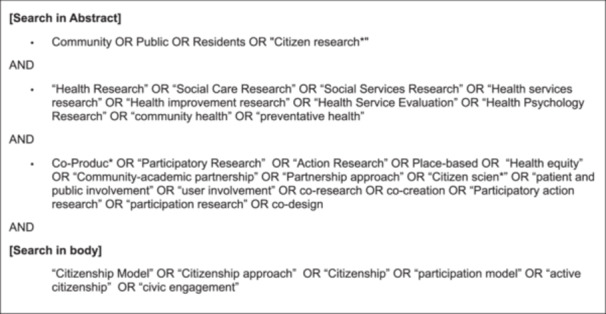
Keyword search strategy.

A subject‐specialist University librarian supported authors in identifying relevant electronic databases. Authors (L.D., C.R.) developed a keyword search strategy using the population, context and concept mnemonic [25]. This resulted in the development of four distinct search strings, which were combined with the Boolean AND function (Figure 1). Search terms were identified from relevant literature and discussion among the whole research team. Search terms were synonyms or near‐synonyms to key terms that defined the population, context, and concept. The first search string defined the *population* as ‘community’, the second string defined the *context* as ‘health research’, and the third string defined the *concept* as ‘co‐production’. It was recognised that some researchers may engage in co‐production but use different terminology, and that the range of potential research methods is wide. To accommodate this, the third string contained terms that were synonymous with or strongly indicative of approaches involving co‐production, as identified through background research and the research team's own experience with co‐production. A fourth ‘concept’ search string was developed to identify studies referencing participatory citizenship models. Due to the recent, rapid increase in co‐production, and the relatively low number of articles identified in the initial screening, no additional limits (e.g., date parameters) were placed on the electronic database search, which was undertaken January–April 2024.

### 2.5 Screening

Articles identified from the database search were uploaded into Rayyan.ai software [26], which was used to remove duplicate articles. A two‐stage screening process was undertaken by three authors (L.D., C.R., J.H.). Stage one: authors (L.D., C.R., J.H.) independently screened titles and abstracts of all retrieved articles using Rayyan.ai [26] to ensure blinding. Following initial screening, all convened and removed the blinding to discuss decisions. Disagreements were resolved by consulting the experienced fourth researcher (E.B.), who was not involved in the initial screening. At this stage, 33 articles remained, and full texts for all articles were retrieved for further assessment. Stage two: five authors (C.R., J.H., M.L., K.A., M.N.) each independently read and screened all articles against the inclusion and exclusion criteria (Table 1). Papers were included if the reported study was underpinned by any participatory citizenship model, and co‐production was the primary methodology. No parameters were put on the type of co‐production methodology included (e.g., qualitative, observational, creative). Only peer‐reviewed studies were included to increase the likelihood of methodological rigour and clear theoretical underpinnings. Given the pragmatic and ethical differences in research conducted with children and adults, only studies reporting research undertaken with adults were included to increase the comparability and transferability of findings. All authors convened to discuss and resolve decisions together.

### 2.6 Data Extraction and Synthesis

#### 2.6.1 Extraction of Key Characteristics

Authors (C.R., J.H.) extracted key characteristics from each study: *authors, country of origin, publication date, design, methods, characteristics of the co‐production group*, and *underpinning citizenship model*.

#### 2.6.2 Thematic Synthesis

Following the extraction of key characteristics, authors (C.R., J.H.) read through each article and accompanying tables and figures to extract all material related to co‐production in health research. This formed a data set that was subsequently analysed using a thematic synthesis technique based on the methods outlined by Thomas and Harden [27]. The analysis began with CR and JH coding the data set line‐by‐line. Codes were applied to data sections (individual words, sentences, or sections) that responded to the research question: *How do participatory citizenship models inform co‐production practice in health research?* Codes were applied to extracted data and recorded on Microsoft Word tables. Following coding, authors (C.R., J.H.) worked independently across the data set, clustering codes similar in meaning or related to similar phenomena. These created initial themes describing relevant aspects of co‐production related to participatory citizenship models. Working together, the authors grouped related themes into a set of overarching themes that defined key patterns or relationships in the data. The research team then discussed themes, developing an understanding of each theme's potential new conceptual underpinning, refining their understanding and definitions of the themes, and creating shared and extended understandings of the breadth and detail of each one. Analysis ended when the research team was satisfied that the themes answered the research question.

## 3 Findings

**FIGURE 2:**
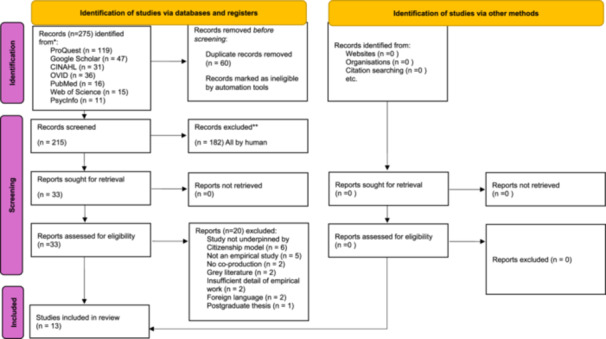
PRISMA 2020 Flow diagram for new systematic reviews, which included searches of databases, registers and other sources. *Consider, if feasible to do so, reporting the number of records identified from each database or register searched (rather than the total number across all databases/registers). **If automation tools were used, indicate how many records were excluded by a human and how many were excluded by automation tools. *From:* Page MJ, McKenzie JE, Bossuyt PM, Boutron I, Hoffmann TC, Mulrow CD, et al. The PRISMA 2020 statement: an updated guideline for reporting systematic reviews. BMJ 2021;372:n71. doi: 10.1136/bmj.n71. For more information, visit: http://www.prisma-statement.org.

The search and screening process is documented in a PRISMA [23] flow‐diagram (Figure 2).

### 3.1 Included Studies

Data from 13 original studies were included in the synthesis, reflecting co‐production with >627 adults engaged in health research (Table 2). Publication dates ranged from 2009 to 2023. Seven studies were published ≤5 years ago, and only two were published >10 years ago. Studies were drawn from Europe and North America. European studies came from the UK [19, 28, 29, 30] (*n* = 4), Netherlands [28] (*n* = 1) and Spain [32] (*n* = 1). North American studies from Canada [33, 34, 35] (*n* = 3), USA [36, 37, 38] (*n* = 3), and one North American study covered research in both Americas [39].

**TABLE 2:** Studies included in the review.

	Authors, date, country	Research area	Design	Methods	Study sample: Characteristics	Co‐production group:Characteristics and recruitment
1	Cogan et al. (2021) [19], Scotland	Mental health	Qualitative	Focus group study	*N* = 40 citizens	*N* = 6 Peer Researchers.
					**Community:** Citizens with lived experience of mental health problems, and major life disruption ≤5 years ago.	**Community:** Citizens recruited from the University Service User and Carer Network (with lived experience of mental health problems)
2	Dupuis et al. (2016) [33], Canada	Dementia	Qualitative	Creative arts workshops	*As co‐production group*	*N* = 8 citizens with dementia
						*N* = 7 family members
						*N* = 15 visual/performance artists
						**Community:** Citizens with lived experience of dementia were recruited from local Alzheimer Societies
3	Foster et al. (2021) [28], UK	Homelessness and problem substance use	Mixed‐methods feasibility study	Qualitative interviews; Peer‐navigator interviews; observations and quantitative outcomes measures*	*N* = 68 (intervention group)	*N* = 2 Study Steering group
						*N* = 4 Peer Navigators*
					**Community:** citizens experiencing homelessness and problem substance use	
						*N* = 6 Citizens from a PPI Group
						*N* = 8 Peer researchers recruited from the Scottish Drugs Forum*
						**Community:** citizens with lived experience of homelessness and problem substance use were recruited through existing services and forums working with these communities.
4	Gignac et al. (2022) [32], Spain	Air pollution	Mixed‐methods	Online surveys; workshop	*N* = 488 (citizen survey 1)	*N* = 40 (community meeting)
						*N* = 50 (co‐creation workshop)
					*N* = 556 (citizen survey 2)	**Community:** General population of the region.
					*N* = 1200 (pop‐up intervention, canvas activity)	
					**Community:** General population (no specific health community)	
5	Grootjans et al. (2022) [28], Netherlands	Health inequalities	Mixed‐methods	Interviews, observations	*N* = 49 interviewees (residents)	*N* = 13 residents of residential areas studied, average age 65 (35–81); 8% males; 33% non‐Western immigrant. 62% in residential area ≥5 years.
					**Community:** Residents in deprived neighbourhoods	
						**Community:** Residents recruited through neighbourhood contacts, snowball sampling and community advertising.
6	Komporozos‐Athanasiou et al. (2018) [29], UK	‐ Cancer Research; ‐ Stroke Research; ‐ Pre‐term Birth	Mixed‐methods	Interviews, ethnography	*As co‐production group*	*N* = 60 Citizens from a Cancer Forum**
						*N* = 20 Citizens from a Stroke Forum **
						*N* = 23 Citizens from a Pre‐Term Birth Forum**
						**Communities:** Lived‐experience of physical/neonatal health issues recruited throughexisting forums (above)
7	La Scala et al. (2023) [36], USA	Health inequalities	Qualitative	Discussion sessions	*As co‐production group*	*N* = 8 Citizen Advisory Board comprised from the local community of Latina mothers
						**Community:** Latina community living in an area of deprivation, who had all previously participated in University research undertaken by the authors' department.
8	Richardson et al. (2023) [34], Canada	Health inequalities	Qualitative	Community consultations, group and 1:1 discussion	*As co‐production group*	Participant numbers not reported. Participants recruited were from six minority (anglophone) communities across Quebec.
						**Community:** Anglophone minority communities across Quebec recruited with support from local stakeholders and community organisations.
9	Schneider (2012) [35], Canada	Schizophrenia	Qualitative	Interviews and Focus Groups	*N* = 11 interviewees (citizens with lived experience) (study 1)	*N* = 7 Citizens with lived experience (study 1)
					*N* = 28 (citizens with lived experience) (study 2)	*N* = 8 Citizens with lived experience (study 2)
					**Community:** Citizens with lived‐experience of Schizophrenia	**Community:** Citizens with lived‐experience recruited through the Schizophrenia Society and a local support group.
10	Starling & Tanswell (2018) [30], UK	Health inequalities	Qualitative	Interviews and creative methods	*As co‐production group*	*N* = 25 young adults (aged 19–30+). 52% male. 44% non‐white British ethnicity.
						**Community:** Young adults not currently in employment, education or training from urban area. Recruited through local statutory and charitable organisations working with this community.
11	Wallersteinet al. (2017) [39], USA and Brazil	Health inequalities	Mixed methods	Listening surveys, focus groups, observations (Project 1);	*Not reported*	*N* = 311 Peer Research Fellows; 125 community teams (Project: Healthy Native Communities Fellowship);
				Educative fieldwork; Discussion groups; multimedia creative methods (Project 2)		
						Participant numbers not reported (Amazonian Project – 2700 inhabitants)
						Participant numbers not reported (Project ‐ Capela do Socorro)
				Systematisation of Experiences methodology; leadership mapping (Project 3)		
						**Communities:** Native communities recruited through local statutory, charitable and community organisations working with these communities.
12	Alang et al. (2021) [37], USA	Health inequalities	Qualitative	Discussion and consultation	*Not reported*	Participant numbers not reported.
						**Communities:** Marginalised citizens with a range of complex health and social needs recruited with support from local stakeholders, community connectors and citizens.
13	Berge et al. (2009) [38], USA	Diabetes	Mixed methods	Intervention design; group discussions; health outcomes measures	*N* = 40 individuals with diabetes and family supporters (pilot study data)	Six adolescents with Type 2 diabetes and their family supporters (Study 1)
						American Indian Families managing diabetes well (numbers unreported) – create an Action Planning Group.
					**Communities:** American Indian families in which an adolescent is living with Type 2 Diabetes.	
						**Communities:** American Indian families in which an adolescent is living with Type 2 Diabetes recruited through a local health care clinic.

* Reported in Parkes et al. 2022. doi: 10.3310/WVVL4786.

** Fora membership figures are approximate and reported in Komporozos‐Athanasiou et al. 2018.

### 3.2 Areas of Health Research

Table 2 shows the health research areas studies addressed. Seven focused on a single area: mental health [19] (*n* = 1), dementia [33] (*n* = 1), homelessness and problem substance use [28] (*n* = 1), air pollution [32] (*n* = 1), neuroscience [36] (*n* = 1), schizophrenia [35] (*n* = 1), and diabetes [38] (*n* = 1). One study included people with lived‐experience of different conditions [29] (cancer, stroke, and pre‐term birth). Six studies explored health inequalities [30, 31, 34, 36, 37, 39]. Table 3 lists the health research areas covered in order of publication, showing that health inequalities were a focus of research undertaken in the last 5–10 years, and that research has increasingly focused on issues affecting broad population groups (e.g., *‘air pollution’*, *‘health inequalities’*). This contrasted with the earliest studies, which focused on discrete health conditions.

**TABLE 3:** The research focus, methods, and underpinning theoretical models of the included studies in order of publication date.

	Date*	Authors	Country	Areas of health	Methods	Theoretical models underpinning the research
≤5 years	2023	La Scala et al. [36]	USA	Health inequalities	Qualitative (Group discussions)	Community‐Based Participatory Research (CBPR)
	2023	Richardson et al. [34]	Canada	Health inequalities	Qualitative (Community consultation)	Community‐Based Participatory Research (CBPR)
	2022	Gignac et al. [32]	Spain	Air pollution	Mixed methods (Online surveys; community workshops)	Citizen Science
	2022	Grootjans et al. [28]	Netherlands	Health inequalities	Mixed‐methods (interviews, observations)	Citizen Science
	2021	Cogan et al. [19]	UK	Mental health	Qualitative (Focus groups)	Rowe's 5R's of Citizenship and Community‐Based Participatory Research (CBPR)
	2021	Foster et al. [28]	UK	Homelessness and problem substance use	Mixed‐methods (interviews; outcomes measures)	Community‐Based Participatory Research (CBPR)
	2021	Alang et al. [37]	USA	Health inequalities	Qualitative (Community Consultation; discussion groups)	Community‐Based Participatory Research (CBPR)
6‐10 years	2018	Komporozos‐Athanasiou et al. [29]	UK	Physical health services (mixed)	Mixed‐methods (interviews, ethnography)	Health Citizenship
	2018	Starling and Tanswell [30]	UK	Health inequalities	Mixed‐methods (Creative methods; interviews)	Cultural Democracy
	2017	Wallerstein et al.	USA	Health inequalities	Mixed methods (listening surveys, focus groups, observations)	Community‐Based Participatory Research (CBPR)
	2016	Dupuis et al. [33]	Canada	Dementia	Creative arts group	Narrative Citizenship
>10 years	2012	Schneider [35]	Canada	Schizophrenia	Qualitative (interviews, focus groups)	Community‐Based Participatory Research (CBPR)
	2009	Berge et al. [38]	USA	Diabetes	Mixed‐methods (group discussion, outcomes measures)	Community‐Based Participatory Research (CBPR)

* From time of the current systematic review was conducted (2024)

### 3.3 Co‐production Methods

Most studies utilised a designated co‐production group, ranging in size from 6 to 103 citizens (median 18 citizens). Samples were generally small (median 45 citizens; range 39–2244). Designs were qualitative [19, 30, 33, 34, 35, 36, 37] (seven studies) or mixed‐methods [28, 29, 31, 32, 38, 39] (six studies). Twelve studies (92%) included traditional qualitative research methods, these being interviews, focus groups, discussion groups, consultation or workshop sessions [19, 28, 29, 30, 31, 32, 34, 35, 36, 37, 38, 39]. Four studies (31%) used observational/ethnographic methods [28, 29, 31, 39] and two (23%) used creative methods [30, 33]. Table 3 shows studies published ≤5 years ago were co‐produced using open community and stakeholder consultation events and workshops, contrasting with the focused recruitment of earlier studies.

### 3.4 Underpinning Citizenship Models

Table 3 shows that community‐based participatory research (CBPR) was the most frequent underpinning model, mentioned in eight articles [19, 28, 34, 35, 36, 37, 38, 39] and spanning the data set's timeframe. Studies underpinned by CBPR originated in North America or the UK, contrasting with the two articles from mainland Europe underpinned by Citizen Science [31, 32]. One UK article was underpinned by both CBPR and Rowe's 5R's model [19] and another UK article was underpinned by Health Citizenship [29]. Both articles using creative methods were underpinned by citizenship models related to creative expression (Cultural Democracy [30]; Narrative Citizenship) [33]. Table 4 details the Citizenship models underpinning the included studies.

**TABLE 4:** Citizenship models underpinning included studies.

Model name	Core principles	Role of citizens	Strengths	Limitations/challenges	Key theorists
Community‐based Participatory Research (CBPR)	Collaboration; Partnership; Trust; Equity; Diversity and Inclusion; social justice; mutual learning; knowledge‐democracy, egalitarianism; shared‐power; shared‐ownership; community action	Collaborative working with researchers; partnership‐building; sharing lived experience and place‐based knowledge; sharing decision‐making, action and ownership; community development;	Generates ‘deep’ place‐based, contextualised knowledge; increases acceptability and applicability of research to community of interest; supports community development, action and capacity‐building; builds trust between communities and academia; supports inclusion and social justice; empowers individuals and communities.	Dominance of positivist scientific‐paradigm; Resource‐intensive methods; evaluation of outcomes; potential for low‐transferability; fair designation of responsibility; unequal capacities and knowledge of stakeholders; academic hegemony; ability to accommodate individuals′ needs; building and maintaining authentic relationships; continuation and legacy work [37, 40, 41]	Kurt Lewin [42]
					Paulo Freire [43]
					Nina Wallerstein [39]
					Fals Borda [44]
Co‐created Citizen Science	Collaboration; Partnership‐working knowledge‐exchange; scientific investigation; problem‐solving [32, 45]	Collaborative working with scientists; Varying levels of input from supplying data to co‐creation [32, 45, 46]	Increases capacity for data collection; enables access to larger datasets; increases representativeness of data; improves interpretation of data; develops trust between communities and scientists; improves the acceptability and applicability of findings/interventions to communities; increases scientific literacy and interest in the public; develops citizens' scientific and research skills and confidence; empowers lay scientists to contribute to knowledge; opportunity to help others through science.^59–619,10^	Public engagement; maximising representation; academic control/hegemony; distrust or misinformation about science; data‐security and ethical data‐sharing; quality control; does not necessarily lead to community action or to change benefitting the communities involved [32, 45, 46, 47]	Alan Irwin [46, 47]
					Muki Haklay [48]
					Rick Bonnay [49]
5R′s of Citizenship	Inclusion; social justice	Citizenship is defined by the extent of people′s connection to the 5 R′s ‐ their Rights; Responsibilities; Roles; Resources; Relationships.	Highlights areas where people experiencing mental health issues face barriers to citizenship; supports inclusion and social justice; holistic psychosocial conceptualisation of citizenship [19, 50, 51].	Dependent on access to sufficient relationships and social opportunities; some people will require extensive support to connect with all five R′s [19, 50]	Michael Rowe [51, 52, 53]
Health Citizenship	Emancipation; Autonomy; Empowerment; Equity; transparency; shared‐knowledge; shared decision‐making; social justice; activism [54]	Shared‐knowledge; shared decision‐making; health activism [54]	Enhances ethical medical practice; supports social justice; emancipates both medical staff and patients from historically oppressive practice; generates trust between patients and meical professionals; support a patient‐centred model of care [54].	Conflict and resistance between healthcare staff and patient groups; change takes time; many patients are non‐activists; power‐imbalance between healthcare staff and patients; tokenistic and limited patient involvement activities [29, 54]	Charlotte Williamson [54, 55]
Cultural Democracy	Creativity; creative contribution; community creativity; democracy; equity; creative participation; creative freedom; creative hope	Access to cultural and creative activities and creative output within local communities; access to creative opportunities, choices and creative resources	Promotes both amateur and professional creative activity; supports self‐expression; promotes hope and flourishing at individual and community level; promotes increased cultural capacity, promotes equitable distribution of creative resources and opportunities; promotes development of a creative ecosystem;	Public engagement in creative and cultural activities; investment of resources; meeting diverse needs and preferences to promote inclusion and equity.	Jonathan Gross [56, 57]
					Nick Wilson [57]
					Kevin Mulcahy [58]
					Owen Kelly [59]
Narrative Citizenship	Autonomy, agency, creativity, relational agency, advocacy, inclusion, social justice, activism [33, 60]. Baldwin; Dupuis	Citizenship is enacted through the autonomous narration of personal stories and through contribution to and identification with collective narratives. Narratives define citizenship, and dominant narratives can be challenged by individuals and communities' own stories; new narratives can be created and recreated to develop inclusivity and diversity [33, 60] Baldwin; Dupuis	Promotes inclusion; challenges stereotypes and stigma; develops empathy; increases self‐expression; provides opportunity for shared‐learning; opportunities for activism; opportunities for personal growth [33, 60] Baldwin; Dupuis	May be resource intensive (e.g., interpersonal work; creative resources); may challenge hegemonic discourse; may be emotionally intense or challenging.	Clive Baldwin [60]

### 3.5 Findings From Thematic Synthesis

**FIGURE 3:**
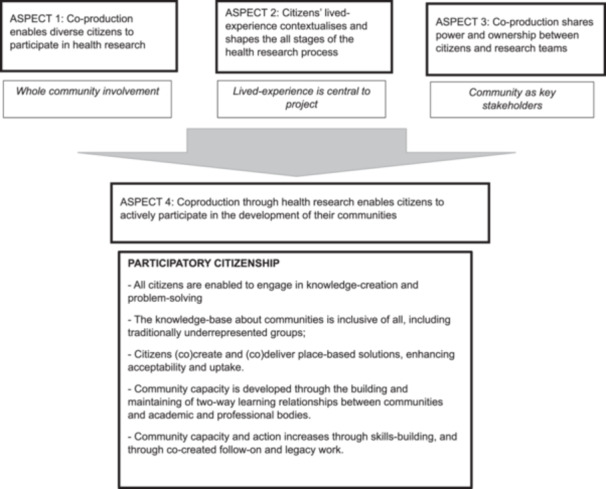
How co‐production in health research enables participatory citizenship.

Thematic synthesis identified four aspects characteristic of how participatory citizenship models informed co‐production in health research (Table 5). These were: Aspect (1) *co‐production enables diverse citizens to participate in health research*; Aspect (2) Citizens' *lived‐experience contextualises and shapes the health research process*; Aspect (3) *Co‐production shares power between citizens and research teams*; and Aspect (4) *Co‐production through health research enables citizens to actively participate in the development of their communities*. Table 6 shows the contribution of each article to these aspects. All studies contributed, and most extensively, to aspects 1–3. Over three‐quarters explicitly contributed to aspect 4. Figure 3 shows the relationship between the identified aspects and how aspects 1–3 contribute to aspect 4.

**TABLE 5:** Findings of the thematic synthesis: Four aspects characteristic of how participatory citizenship models inform co‐production in health research.

Aspect 1: Co‐production enables diverse citizens to participate in health research.	Citizenship‐informed research enabled a diverse range of citizens to participate, and many studies specifically targeted and supported participation of marginalised and underrepresented communities. Inclusive research methods aimed to overcome barriers to participation. These methods included flexible and iterative research designs, flexible administrative and delivery processes, use of community‐based research methods, operation from community venues, working in partnership with trusted organisations, use of non‐traditional and creative research methods, use of qualitative or observational methods, working with peer supporters, and the provision of support and training for those engaged in co‐production by the professional research team throughout the process.
Aspect 2: Citizens' lived‐experience contextualises and shapes all stages of the health research process.	Citizens lived‐experience made a central contribution to citizenship‐informed health research. All members of the co‐production group were valued for the diverse perspectives, narratives, and creative expression they were able to contribute. Citizenship‐informed co‐produced research was undertaken and understood within the context of the community of interest, including that community′s lived experience of health and research, its history, places and local values, beliefs, and structures.
Aspect 3: Co‐production shares power between citizens and research teams.	Citizenship‐informed research practice enabled power to be shared between citizens and research teams authentically, and the traditional hierarchy between public and professional contributors to be levelled. Studies shared power in various ways at various stages of the research process, including during the setting of the research agenda and research questions, the research design, development of findings, dissemination, ongoing work and co‐ownership of the project.
Aspect 4: Co‐production through health research enables citizens to actively participate in the development of their communities.	Studies showed that citizenship‐informed research practice was able to transform health research through the introduction of new perspectives, including from citizens previously underrepresented by health research. Some studies reported how engagement with research had enabled citizens to increase their engagement with their community, including through continued research, political activity, work to improve health and wellbeing in their communities, and through increased engagement with education or employment.

**TABLE 6:** Contribution of the included articles on each of the four aspects.

Aspect	1	2	3	4	5	6	7	8	9	10	11	12	13
Aspect 1: Co‐production enables diverse citizens to participate in health research.	**	**	**	*	**	**	**	*	**	**	**	**	**
Aspect 2: Citizens' lived‐experience contextualises and shapes all stages of the health research process.	**	**	**	*	**	**	**	**	**	**	**	**	**
Aspect 3: Co‐production shares power between citizens and research teams.	**	**	**	*	*	**	**	*	**	**	**	**	**
Aspect 4: Co‐production through health research enables citizens to actively participate in the development of their communities.	—	**	**	—	**	**	**	—	**	**	**	**	**

*Note:* 1: Cogan et al. (2021); 2: Dupuis et al. (2016); 3: Foster et al. (2021); 4: Gignac et al. (2022); 5: Grootjans et al. (2022); 6: Komporozos?Athanasiou et al. (2018); 7: La Scala et al. (2023); 8: Richardson et al. (2023); 9: Schneider (2012); 10: Starling & Tanswell (2018); 11: Wallerstein et al. (2017); 12: Alang et al. (2021); 13: Berge et al. (2009); —: not a focus.

* Some focus.

** Extensive focus.

#### 3.5.1 Aspect 1: Co‐production Enables Diverse Citizens to Participate in Health Research

Citizenship‐informed co‐produced studies were inclusive and most included participants from underrepresented communities. Eight authors co‐produced with communities at risk of experiencing health inequalities who were not already members of service user research groups, forums, or databases [28, 30, 31, 33, 34, 35, 37, 38, 39]. These included residents living in deprived neighbourhoods [28], those with complex health needs [28, 37], ethnic minorities [33, 34] and young adults not engaged in employment or education [30]. Recruitment from underrepresented communities was usually community‐based, requiring planning, creativity and local connections. Table 2 shows the range of formal and informal contacts used to support recruitment.

Although co‐production groups were frequently small in size, most authors reported that citizenship‐informed co‐production involved considerable time and resources, including activities such as regular Patient and Public Involvement (PPI) groups [28, 35] or creative projects [30, 33]. Researchers described co‐production as *‘iterative’* [34], *‘slow and messy’* [38], *‘Nonlinear’* [30] and its management as *‘embracing the chaos’* [30]. Citizens required support and training to fully engage in co‐production, and work with marginalised communities often ran over extended periods of time [28, 30, 31, 35, 38, 39]. Starling and Tanswell [26] described the management of frequent unplanned absences. and having to design activities that could be completed in one session. Foster *et al* [28] adjusted academic language that citizens found alienating or triggering. In contrast, two studies ran larger‐scale one–off community activities engaging more citizens but not specifically targeting or supporting underrepresented groups [32, 34].

Community trust was central to success, and authors used various approaches to trust‐building, with community stakeholders often serving as advocates or gatekeepers [28, 29, 30, 31, 33, 34, 35, 37, 39]. Financial resources mitigated barriers like ‘out‐of‐pocket’ expenses, which particularly impacted citizens on low incomes, and Foster et al. [28] described the difficulty in negotiating University finance systems to reimburse citizens' expenses. Some studies convened a citizen‐led advisory group to oversee the research project from start to finish [28, 38]. Research teams drew on citizens' place‐based knowledge to design studies that were accessible and culturally appropriate for communities [36, 38, 39]. Citizens often undertook data collection, being trusted by their community and understanding local perspectives [28, 31, 35, 38, 39]. Some studies provided ongoing opportunities for citizens after the research ended [30, 35], which Foster et al. (2021) suggest prevented participants feeling ‘*dropped’*, a particular risk for citizens who have experienced marginalisation. Ongoing opportunities also enabled participants to learn, build skills and make further contributions to their community beyond the original research project (Table 7).

**TABLE 7:** Activities of the co‐production group.

ID	Authors, date, country	Co‐production group size	Co‐production group activities	Co‐production group training and support	Ongoing contact/contribution
1	Cogan et al. (2021) [19], Scotland	*N* = 6	‐ Involvement in all stages of the research process, including data collection, analysis, developing conclusions, project steering.	‐ Support of experienced researchers	‐ Group were already citizens involved in a University‐based PPI group
2	Dupuis et al. (2016) [33], Canada	*N* = 15 (citizens with lived‐experience)	‐ Creative research data/output	‐ One‐day creative workshop facilitated by professional artists and research team.	‐ None reported
		*N* = 15 visual/performance artists	‐Dissemination through performance and media		
3	Foster et al. (2021) [28], UK	*N* = 20	‐ Involvement in all stages of the research process, including design of study materials, data collection, analysis, and dissemination	‐ Administrative support by the research team	‐ Research team remained in touch
					‐ Co‐authorship of academic papers
4	Gignac et al. (2022) [32], Spain	*N* = 90	‐ Co‐design of the research question and study protocol	‐ Facilitating activities	‐ Project described as ‘ongoing’, though
				‐ Providing an assessment of the viability and feasibility of ideas	involvement levels unclear
5	Grootjans et al. (2022) [28], Netherlands	*N* = 13	‐ Citizen scientists were involved in data collection, data analysis and dissemination	‐ Two × 2.5 h citizen science training sessions.	‐ Citizen science meetings
6	Komporozos‐Athanasiou et al. (2018) [29], UK	*N* = 103	‐ Forums engaged citizen members in strategic discussion about research policy and studies planned and the preparation of grant applications.	‐ Three separate external health forums whose citizens attended regular formal research meetings set up and facilitated by professional research teams.	‐ Ongoing engagement with health forums
7	La Scala et al. (2023) [36], USA	*N* = 8	‐ Citizens advise the research team on how to engage and recruit their community in research, and how the research institution could support the community.	‐ Community Advisory Board meetings set up and facilitated by the professional research team.	‐ Biannual newsletter
					‐ Bank of free/low‐cost mental and physical health resources regularly updated and available to the community
8	Richardson et al. (2023) [34], Canada	Not reported	‐ Community stakeholders and organisations involved in selection of target communities, data collection events, analysing and disseminating findings.	‐ Community discussion participants did not receive training	‐ None
9	Schneider (2012) [35], Canada	*N* = 15	‐ Involvement throughout the research, including proposal and grant application, data collection and analysis, dissemination and legacy work	‐ Research skills training and group facilitation provided by professional research team	‐ Citizens continue to engage in ongoing dissemination activities
10	Starling & Tanswell (2018) [30], UK	*N* = 25	‐ Dissemination of research findings through creative output	‐ Series of creative sessions, exploring science, creative media production, and cultural field‐trips. Facilitated by science and creative professionals and the research team.	‐ Ongoing partnership with research team
					‐ Continued career/educational development for citizens involved with the project
11	Wallerstein et al. (2017) [39], USA and Brazil	*N* = 311	‐ Citizens involved in all stages of the research including planning, data collection, analysis and dissemination.	‐ Health research and community leadership training programme (HNCF) for native community citizens. A year‐long process that includes retreats, internet‐based contact, and practical support from community mentors, programme facilitators and professional researchers.	‐ Ongoing work, but survival of the HNCF project is challenged by precarious project funding
				‐ education in research topics (e.g. sanitation)	
				‐ training in participatory methods (e.g. photovoice).	
12	Alang et al. (2021) [37], USA	Not reported	‐ Citizens involved at all stages in identifying the research problem, in data collection, in reviewing findings.	None described	‐ Informal ongoing contact with citizens beyond project work sometimes maintained
13	Berge et al. (2009) [38], USA	*N* = 6	‐ Citizens were involved in all stages of the research, including the development of the intervention, design of the research study, data collection, analysis and dissemination	Support from healthcare providers and researchers	‐ Those initially involved in the project are providing training and support to new community support partners

#### 3.5.2 Aspect 2: Citizens' Lived‐Experience Contextualises and Shapes All Stages of the Health Research Process

Authors of the included studies valued lived‐experience and placed this at the centre of the research process. Non‐linear, iterative and place‐based co‐production generated contextualised, pluralistic knowledge, and qualitative, observational and creative methods effectively communicated citizens′ perspectives and life experiences. If used at all, quantitative methods were components within mixed‐methods studies (Table 2). Several studies show that citizenship‐informed co‐production effectively supports the translation of health research to real‐world practice, and enables citizens to feel they have made positive civic contributions [38, 39]. For example, Berge, Mendenhall and Doherty [38] co‐produced a diabetes self‐management programme with adolescents with diabetes who reported feeling valued through contributing their lived‐expertise. In other studies, citizens applied local knowledge and lived‐experience to engage their community in health research and identify the health issues affecting local people [28, 30, 33, 35, 36, 37].

Across studies, citizens engaged in multiple aspects of research, except for administration or financial governance (Table 7). Research teams provided direction, hands‐on support and training where citizens undertook technical tasks, such as data analysis [19, 28, 31, 34, 35, 37, 38, 39]. Some authors did not involve citizens in technical analysis at all [29, 33] or did not report this [30, 32, 36]. Eight studies reported citizens' engagement in dissemination [28, 30, 31, 33, 35, 38, 39], including creative output [33], multi‐media dissemination of lay findings [28, 30], presentations to policymakers [28], and co‐authorship of academic conference papers [35, 37]. Less commonly, citizens selected the research topic or question [30, 32, 35, 37, 39].

Where co‐production continued beyond the end of the original research, the sustainability and influence of health research on community health policy and outcomes often increased. Legacy work included ongoing research group contact [28, 30, 37], dissemination [28, 35], new grant applications and projects [19, 39], and regular co‐production group meetings or meetings as a wider forum [19, 29, 31, 39] (Table 7). However, Wallerstein et al. [39] described how short‐term funding threatened ongoing co‐production, citizen engagement and community development, which requires continued investment of resources.

#### 3.5.3 Aspect 3: Co‐production Shares Power **B**etween Citizens and Research Teams

Studies levelled the power distribution between researchers and communities, and the extent of this levelling was largely determined by the amount and type of contribution citizens made relative to the professional team. Schneider [35] described a research project successfully co‐produced at all stages, but highlights the skewing of power that occurred when the grant was awarded to the lead investigator rather than the co‐production group, giving academics financial and administrative control. Komporozos‐Athanazious et al. [29] described how formalised, mandated PPI processes created structured and ritualistic, rather than authentic, co‐production. Their ethnographic study observed PPI meetings in health settings controlled by professionals through formal meeting structures, and the emancipatory actions of citizens who challenged (dominant) professional discourses.

Several studies described processes undertaken to increase awareness of factors creating inequity and division in the research. La Scala et al. [36] created positionality maps for team members to render visible the shared, differing and intersecting social identities, potentially creating power imbalance or biases. Revealing unconscious biases and power differentials within the research process was sometimes uncomfortable or challenging to researchers: ‘*I take on some of the assumptions […] and have stereotypes that sometimes I don′t even understand that I have and then I go to one of these workshops with people with dementia and I′m reminded about that* [33]*.*’ (p. 368). Alang, Batts and Letcher [37] described three principles as essential to developing equitable research partnerships with communities: ‘*authentic engagement*,’ ‘*defining and living values*’ and ‘*embracing accountability*’. These include actions such as spending time learning about communities' interests (*authentic engagement*), providing monetary (or equivalent) compensation for citizens' contributions (*defining and living values*), and supporting communities to utilise health research to advance social justice and accountability (*embracing accountability*).

Studies demonstrated that knowledge and expertise held by citizens and researchers are not equal in all areas creating power imbalances at different stages of a research project. For example, Gignac et al [32] described how scientists' knowledge of the evidence base, research design and protocol requirements led them to reject some of the citizens' ideas and to exclude citizens' from technical roles. Schneider [35] describes how power dynamics change as citizens are upskilled: *‘[O]ne cannot simply “give” people power; they must also be ready and willing to take it up[…] power is a constantly shifting resource requiring attention not just at the beginning of projects but throughout*. (p. 160)’ Several studies showed that co‐production enabled citizens to challenge accepted ‘truths′ by introducing new perspectives. In a project underpinned by a narrative citizenship model, Dupuis [33] engaged people with dementia in a creative project that created a counter‐narrative to the dominant ‘tragedy’ narrative about dementia. Similarly, Schneider [35] describes the impact of research co‐produced with mental health service users on their doctor, who was unused to hearing former patients speak *‘eloquently and poignantly’* and who changed their subsequent interactions with service users in response. Many participatory citizenship models highlight the need for shared power as citizens shape the civic society they are part of through their contributions. The current findings demonstrate that shared power and levelling relational hierarchies are key aspects of authentic citizenship‐informed co‐production.

#### 3.5.4 Aspect 4: Co‐Production Through Health Research Enables Citizens to Actively Participate in the Development of Their Communities

Some authors reported that their own research practice was transformed through citizenship‐informed co‐production. In some studies, citizens were able to transform research language and methods to better‐fit communities [28, 30, 36, 38, 39]. Dupuis [33] writes: *‘It will make me think differently about how I do research with some marginalised communities’* (p. 370). Individual and community health and well‐being was transformed through co‐production, shown in outcomes like increased access to employment and education opportunities [28, 30, 39], further engagement in research [28, 29, 35], increased understanding and engagement with local health issues [31, 37, 39], continued work to reduce the local health inequalities [37, 38, 39], and ongoing community health activism [29, 31, 33, 35]. Overall, few studies discussed the impact of co‐production on health policymaking, though some groups undertook early, tactical engagement with stakeholders through the project [34, 38] or disseminated findings to policymakers [30, 31, 35, 39].

Figure 3. depicts how the findings of the synthesis connect to show how co‐production in health research, when underpinned by a citizenship model, enables citizens to actively engage in the development of their communities. The diagram shows that aspects 1‐3 enable the inclusion of all social groups, the valuing of citizens' lived experience and give citizens' power within, and ownership of, health research, and how in combination these aspects support active civic participation (Figure 3). The current authors have developed a set of recommendations based on the above findings which are intended to support research teams engaging in a citizenship‐informed co‐production in health research (see Box 1).

Box 1 **Recommendations for practitioners engaging in citizenship‐informed co‐production in health research.**

**Table:** 

*Recommendation 1*	Ensure research methods are inclusive of diverse citizens Identify underrepresented groups and known barriers to participation Consider using non‐traditional research methods Consider where citizens require additional support to co‐produce, and how this could be provided Consider working with trusted community stakeholders and other organisations
*Recommendation 2*	Ensure citizens' lived‐experience and community knowledge are central to the research Informing the research design and methods Informing data analysis and interpretation
*Recommendation 3*	Ensure power is shared between professionals and citizens.
	*Consider* Setting of the research agenda Financial, administrative and creative control Remuneration and compensation Ownership, utilisation and dissemination of findings
*Recommendation 4*	Ensure research has a transformative impact on community health and citizenship Reflect on implications for future health research practice Develop understanding of community health Develop sustainable solutions to health issues Create new opportunities for citizens within health research and wider community life

## 4 Discussion

### 4.1 Increasing Representation in Health Research

This systematic review synthesised evidence on how citizenship models inform co‐production in health research. The findings identified four key aspects of co‐production informed by citizenship models and demonstrate that this approach enables citizens to engage with both health research and community development actively. Included studies were mainly from North America and the UK, concurring with previous research findings [5, 6], and reflecting an increased focus on patient involvement in health research in the UK [4, 61, 62, 63, 64, 65]. The current review synthesised only 13 qualitative papers, meaning that strong conclusions about geographical trends cannot be drawn. However, CBPR models underpinned most papers from North America and the UK, whereas the only two papers identified from mainland Europe were underpinned by Citizen Science models. Future systematic reviews should explore geographical patterns in co‐production, as different geographical regions may underpin co‐production with different theoretical models that have a different relationship (if any) to participatory citizenship.

The low representation of some populations in health research exacerbates health inequalities [66, 67, 68]. The current synthesis found that citizenship‐informed co‐production effectively engaged underrepresented groups. Most included studies were underpinned by citizenship‐models with a strong focus on inclusion and social justice, such as CBPR, Rowe's 5 R's and Narrative Citizenship (Table 4). Notably, one study underpinned by citizen science [32] was effectively co‐produced with a large number of citizens, but did not target or support underrepresented groups. Nevertheless, citizen‐science projects have the potential to involve large numbers of non‐research professionals in the co‐creation of health research on topics of concern to their communities, and in the development of community‐based research‐driven solutions [32].

### 4.2 Flexible, Contextual Research Designs

Our findings showed that flexible, contextual research designs characterised citizenship‐informed co‐production, with researchers working reflexively to identify and reduce barriers, biases, and marginalising terminology. Large‐scale generalisable research designs may fail to identify the place‐based differences underpinning local health inequalities [69]. In contrast, citizenship‐informed co‐production utilises contextual and experiential knowledge to develop a nuanced understanding of community health. Citizenship‐informed co‐production enables communities to understand health issues impacting their communities better, identify and leverage local assets and resources, develop community capacity and co‐design mechanisms of change. The majority, though not all, of the included studies reported on transformative aspects of co‐production, including outcomes for those involved that sustained beyond the original research project.

### 4.3 Active Engagement With Values

Post‐positivist research methods are designed to enable researchers to practice as neutral, value‐free observers and to minimise their influence from research data and analysis (e.g., ‘blinding′ techniques). This approach has been subject to recent debate with increased discussion about the influence of values across scientific research fields, given that researchers all bring their own values to their work regardless of methods applied [70]. As such, there is a role for all researchers to reflect on their position and values in relation to their research, such as through positionality or ‘role as researcher′ statements [36, 71]. Aligning with wider literature on values within research practice, authors have recognised that co‐production is inherently values‐driven [11]. From an analysis of 57 different citations defining co‐production, Masterson and Laidlaw [11] identified 40 values, many of which align with the findings of the current review and are also expressed in participatory citizenship models (Table 4). Masterson and Laidlaw [11] recommend that co‐production groups reflect on and select those values most resonant with their own projects and use these to guide research activity. Similarly, several studies included in the current synthesis describe the efforts of researchers to identify their own values, and those of their community, and the influence of these within the co‐production process.

Studies included in the current review mostly conducted citizenship‐informed co‐production at all stages of the research projects, rather than only in discrete areas. This increased citizens' contribution and ownership of the research and avoided tokenistic involvement. Though democratic principles typically underpin co‐production guidance, criticism is often levelled at projects where co‐production appears shallow or inauthentic [29]. Participatory citizenship models are characterised by values of democracy, action and inclusivity, and so, when used to underpin co‐production, serve to challenge academic hegemony and tokenism.

### 4.4 Investment of Resources

Funding bodies, government and academia traditionally govern health research agendas. Citizenship‐informed research empowers communities to shape the research agenda and investigate questions of importance to them. Co‐produced health research is valuable to stakeholders and policymakers who require knowledge of, and acceptable solutions to, real‐world issues impacting local populations, and which support the reduction of health inequalities. A scoping review by McCarron et al [72] identified that co‐production increases the relevance of research to patients involved in defining research questions and increases citizens' skills, confidence and research knowledge. Further, a large meta‐analysis concluded that co‐creation with citizens improves the efficacy of health interventions [73] Recognition of the value of co‐production by policymakers, funders and stakeholders is demonstrated by the proliferation of, often high‐quality, frameworks and toolkits developed by these bodies which are designed to support researchers and ensure the quality co‐produced research [6, 11]. However, our findings highlighted the financial and human resources required for authentic and inclusive citizenship‐informed co‐production, as researchers need time and funding to build community trust, work flexibly, provide training, support project management, and engage citizens throughout iterative, unpredictable community‐based projects. The investment of adequate resources for small‐scale place‐based research is beyond the provision of many small grants, community organisations, and individuals [69]. A rapid evidence review [74] identified that academic culture and systems create barriers to co‐production, such as researchers' temporary contracts and the competing demands on their time, short‐term funding, and traditional inflexible academic research practices [74]. Policymakers must, therefore, adequately invest in authentic co‐production, informed by participatory citizenship models, and extend this support beyond the release of toolkits and recommendations, useful though these may be. Participatory citizenship models promote broad civic engagement. Investment in co‐production may reap benefits both in terms of health research outcomes (e.g., increased representation and transferability) and in increased civic engagement that extends beyond the parameters of a single health research project.

### 4.5 Psychosocial Investment

The current findings identified the psychosocial demands that citizenship‐informed co‐production can have for research teams. Building equitable partnerships involves sociopolitical engagement with communities that definitively shifts the role of the researcher away from that of a detached, apolitical analyst to that of a fellow citizen engaged in the challenges and ambitions of local communities. Traditional boundaries between researchers and communities are, to an extent, redrawn. As engagement with the experiential reality of communities and reflexive practice reveals hidden power dynamics and biases, researchers may feel confronted, conflicted or defensive. To engage fully in citizenship‐informed co‐production, researchers must reflect on managing social identities, social boundaries and the new relationships that will inevitably develop, end or continue throughout and beyond the project′s lifespan. It is imperative that researchers question their preparedness to undertake a research approach that will level boundaries, involve new methods and challenge familiar roles and identities.

### 4.6 Strengths and Limitations

Three of the four aspects of citizenship‐informed co‐production identified in the current findings were addressed by all included studies, and the fourth aspect was addressed by most. This strengthens the findings, which are thoroughly underpinned by a synthesis of robust, relevant and theoretically driven studies. The model (Figure 3) and recommendations provided by the current authors provide a useful reference point for health researchers engaging in citizenship‐informed co‐production and can guide project evaluation. Further, this model is the first to outline how a theoretical underpinning of participatory citizenship can inform co‐production in health research, and is transferable across different research contexts. Three co‐authors with lived‐experience (M.L., K.A., M.N.) co‐produced multiple stages of this project, including research design, screening, and review of draft findings. They were supported by 2 hour‐long training and discussion sessions run by the authors (C.R., J.H.). A limitation of the study is that, due to the technical and time‐consuming nature of the evidence synthesis, University‐employed authors had greater input in the data analysis and writing‐up stages, which may have created professional bias within the findings. The search strategy was developed with a subject‐specialist academic librarian. However, inclusion of ‘citizenship’ and synonymous terms in the search strategy undoubtedly yielded a smaller data set than might otherwise have been obtained, potentially excluding authors who used citizenship model principles without using explicit citizenship or civic engagement terminology.

## 5 Conclusion

This current synthesis identified four aspects by which participatory citizenship models inform co‐production in health research. Engagement with these aspects supports authentic, inclusive and transformational co‐production. Citizenship‐informed co‐production requires substantial resourcing, planning from the outset and practical and psychosocial commitment from all involved. Nevertheless, underpinning health research with citizenship‐informed co‐production increases knowledge about community health and the development of workable place‐based solutions. Underpinning health research with a participatory citizenship model directs focus outwards, engaging citizens in civic contribution both within and beyond the original project, and avoids shallow, inwardly‐focussed or tokenistic public involvement. The citizenship‐informed approach to co‐production is, therefore, of substantial value to health policymakers and health practitioners who strive to increase representation in health research, reduce health inequalities, and increase civic engagement.

## Author Contributions

**Catharine Rose:** conceptualization, methodology, project administration, investigation, formal analysis, writing ‐ original draft, writing ‐ review and editing. **Lois Donnelly:** methodology, project administration, investigation, writing ‐ original draft, writing ‐ review and editing. **Jess Howdle:** conceptualization, investigation, formal analysis. **Mark Lynes:** conceptualization, investigation. **Katharine Arnett:** conceptualization, investigation. **Mary Nettle:** conceptualization, investigation. **Eleanor Bradley:** conceptualization, methodology, formal analysis, writing ‐ review and editing, supervision.

## Conflicts of Interest

The authors declare no conflicts of interest.

## Patient or Public Contribution

This review was supported by three public contributors (M.L., K.A., M.N.) with lived‐experience of health services, recruited from the University of Worcester IMPACT service user involvement group. They are credited with co‐authorship of this article in recognition of their substantial contribution. All contributed to the research design, independently screened all full‐text articles, reviewed the findings and conclusions, and commented on early drafts.

## Data Availability

The data that support the findings of this study are available from the corresponding author upon reasonable request.
